# Ghrelin expression is associated with a favorable outcome in male breast cancer

**DOI:** 10.1038/s41598-018-31783-x

**Published:** 2018-09-11

**Authors:** Malin Grönberg, Cecilia Nilsson, Ida Markholm, Ingrid Hedenfalk, Carl Blomqvist, Lars Holmberg, Eva Tiensuu Janson, Marie-Louise Fjällskog

**Affiliations:** 1 Department of Medical Sciences, Section of Endocrine Oncology, Uppsala University, Uppsala, Sweden; 2 Center for Clinical Research, Västmanland County Hospital, Västerås, Sweden; 3 Division of Oncology and Pathology, Department of Clinical Sciences, and CREATE Health Strategic Center for Translational Cancer Research, Lund University, Lund, Sweden; 4 Department of Oncology, Helsinki University, Helsinki, Finland; 5 Department of Oncology, Örebro University Hospital, Örebro, Sweden; 6 Department of Surgical Sciences, Uppsala University, Uppsala, Sweden; 7 Faculty of Life Sciences and Medicine, King’s College London, London, United Kingdom

## Abstract

Ghrelin and obestatin are two gastrointestinal peptides, derived from a common precursor. Expression of both peptides have been found in breast cancer tissue and ghrelin has been associated with breast cancer development. Ghrelin expression is associated with longer survival in women diagnosed with invasive and node negative breast cancer. The clinical implications of the peptide expression in male breast cancer are unclear. The aim of this study was to investigate the role and potential clinical value of ghrelin and obestatin in male breast cancer. A tissue microarray of invasive male breast cancer specimens from 197 patients was immunostained with antibodies versus the two peptides. The expression of the peptides was correlated to previously known prognostic factors in breast cancer and to the outcome. No strong correlations were found between ghrelin or obestatin expression and other known prognostic factors. Only ghrelin expression was statistically significantly correlated to breast cancer-specific survival (HR 0.39, 95% CI 0.18–0.83) in univariate analyses and in multivariate models, adjusted for tumor size and node status (HR 0.38, 95% CI 0.17–0.87). HR for obestatin was 0.38 (95% CI 0.11–1.24). Ghrelin is a potential prognostic factor for breast cancer death in male breast cancer. Patients with tumors expressing ghrelin have a 2.5-fold lower risk for breast cancer death than those lacking ghrelin expression. Drugs targeting ghrelin are currently being investigated in clinical studies treating metabolic or nutritional disorders. Ghrelin should be further evaluated in forthcoming studies as a prognostic marker with the aim to be included in decision algorithms.

## Introduction

Male breast cancer (MBC) accounts for approximately 0.7% of all breast cancer cases in the Nordic countries^1^, and 1% in the US^2^. MBC has not been studied as extensively as female breast cancer (FBC) and despite the limited characterization of MBC, patients receive treatment according to guidelines in FBC^3–5^. Whether there is a difference in prognosis compared with FBC is debatable. A poorer outcome has been indicated in several studies^6–8^, but also similar^9,10^ or even better prognosis than FBC^11^ have been reported. Studies on genomic and transcriptional levels have demonstrated molecular differences between MBC and FBC. Gene expression analysis has indicated that MBC tumors can be classified into different intrinsic sub-types luminal M1 and luminal M2 which differ from those established in FBC^12^. Also, significant differences regarding DNA aberrations compared to FBC has been demonstrated using comparative genome hybridization, and a new subgroup unique for male patients was identified^13^. These findings suggest that MBC may be a separate tumor entity from FBC.

Ghrelin is a 28 amino acid peptide hormone, derived from a prohormone, preproghrelin, together with obestatin^14,15^. Ghrelin was identified as a ligand of the growth hormone (GH) secretagogue receptor (GHSR). Ghrelin is produced mainly by gastric oxyntic cells^16^ and is a powerful orexigenic peptide. It is involved in many physiological actions ranging from hormonal secretion, stimulation of appetite, modulation of insulin secretion, adipogenesis and gut motility^14,17–20^. Its involvement in cell proliferation^21–23^ together with the effect on GH levels by the ghrelin axis^14^, gives ghrelin a potential role in tumorigenesis^24^. Another interesting aspect is that low ghrelin levels are correlated to obesity, a known risk factor for breast cancer^25,26^. The C-terminus of proghrelin is processed to give rise to the 23-amino acid peptide hormone obestatin, initially proposed to have opposite roles to those of ghrelin^15^. However, follow-up studies have not been able to confirm these actions and obestatin seem to have independent actions to those of ghrelin although physiological studies are scanty^27–30^.

Several constituents of the ghrelin axis (e.g. ghrelin, obestatin, ghrelin splice variants and GHSRs) have been demonstrated in normal breast tissue, breast tumors and in breast cancer cell lines^31–33^. Ghrelin expression has been associated with a better outcome in different breast cancer studies. Patients with tumors expressing ghrelin have a lower risk for breast cancer death compared to those lacking ghrelin expression in both non-consecutive and selected female patient populations of invasive breast tumors^34^, as well as in a clinically well-characterized case-control study of lymph node negative patients^35^. Also, an increase in breast cancer risk has been shown to be associated with different ghrelin gene polymorphisms^36^. Ghrelin gene-derived splice forms are overexpressed in breast cancer which could suggest that an imbalance in the regulation of the ghrelin system might lead to or influence breast tumor pathogenesis^33,37,38^.

Knowledge about the prognostic impact of many routinely used clinicopathological parameters in MBC is limited and contradictory^39,40^ and since MBC might not be exactly the same disease as FBC, it is important to investigate the role of new potential biomarkers as well as to evaluate how to best assess different biomarkers in MBC.

The aim of this study was to investigate the protein expression of ghrelin and obestatin by immunohistochemical staining of tissue microarrays consisting of 197 invasive breast carcinomas from MBC patients and to assess if any of these peptides could be useful as markers of clinical outcome in this rare disease.

## Materials and Methods

### Patient and tumor characteristics

The National Cancer Register was used to identify male patients with invasive breast cancer diagnosed 1990–2007 in two regions of Sweden (Lund and Uppsala-Örebro), covering a total population of 3.66 million people. Only patients with accessible paraffin-embedded tumor blocks, clinical-pathological data and outcome data were included in the study. Updated information on the patients’ vital status and cause of death was retrieved from the National Population Register. The cohort has been described in a previous study^41^.

Tissue samples from 197 men with breast cancer were included (109 from Uppsala-Örebro and 88 patients from Lund). Clinicopathological characteristics are summarized in Table 1. One-hundred and twenty-four (62.9%) died during the observation period, 41 (21%) related to breast cancer and 83 (42.1%) related to other causes. Mean follow-up time was 54 months (range: 0–180 months). Patients’ characteristics including tumor grade, hormone receptor and HER2 status are shown in Table 1.

**Table 1:** Clinicopathological characteristics.

Variable	n	%
Age (years)	Median 73 (range 23–98)	
Tumour size (mm)	Median 20 (range 4–100)	
**Tumour histology**		
Ductal	170	86.3
Lobular	6	3.0
Other	7	3.6
NA	14	7.1
**Histologic grade**		
I	15	7.6
II	96	48.7
III	81	41.1
NA	5	2.5
**ER**		
Positive	183	92.9
Negative	9	4.6
NA	5	2.5
**PgR**		
Positive	152	77.2
Negative	38	19.3
NA	7	3.6
**HER2 (IHC)**		
0	122	61.9
1	41	20.8
2	19	9.6
3	3	1.5
NA	12	6.1
**Nodal status**		
Positive	77	39.1
Negative	88	44.7
NA	32	16.2
**Vital status**		
Alive	73	37.1
Dead	124	62.9
Dead from breast cancer	41	33.1
**Chemotherapy**		
Yes	21	10.7
No	158	80.2
NA	18	9.1
**Endocrine therapy**		
Yes	119	60.4
No	64	32.5
NA	14	7.1
**Radiotherapy**		
Yes	81	41.1
No	98	49.7
NA	18	9.1

ER, estrogen receptor; HER2, human epidermal growth factor receptor 2; NA, not available; PgR, progesterone receptor.

The study was approved and the need for consent was waived by the local ethics committee, Regionala etikprövningsnämnden (EPN), in Uppsala, Sweden (ref: 2007/254) and Lund, Sweden (ref: 2012/89).

### Tissue microarray construction

Construction of the tissue microarrays (TMAs) has been described elsewhere^41^. Briefly, representative areas from each tumor block were punched and brought into recipient paraffin blocks to construct the TMAs. Two 1 mm cores from each tumor were transferred to the TMA block. From array blocks, 4 µm thick sections were cut and transferred to glass slides.

### Antibodies

The primary antibodies used for immunohistochemical staining were anti-obestatin, in-house developed antibody (rabbit polyclonal), for which the production and characterization has been described previously^42^ and anti-ghrelin (H-031-30, Phoenix Pharmaceuticals, Belmont, CA, USA). Both antibodies were diluted 1:2000.

### Immunohistochemistry

Immunohistochemical staining was performed using the Dako EnVision Plus-HRP Detection Kit (Dako, Glostrup, Denmark) according to the manufacturer’s instructions. For antigen retrieval the sections were subjected to pre-treatment (microwave heating for 10 min at 750 W followed by 15 min at 380 W using Tris-HCl buffered saline, pH 8.0). The sections were incubated with the primary antibodies in PBS with 1% BSA over night at 4 °C. Bound antibodies were visualized by incubation with liquid 3, 3′-Diaminobenzidine substrate chromogen for 5 min.

The analyses of the ghrelin and obestatin immunostainings were manually performed by two observers. None of the investigators had access to clinical data or outcomes. The intensity of the staining in the tumor cells was examined and scored on a scale as non-immunoreactive (non-IR, 0), weak (1), moderate (2) and strong (3), respectively. Each of the two cores from every tumor case on the array was examined and scored separately. In case of conflicting results, a third evaluation was performed and consensus was reached. If one sample from a tumor core was lost, the remaining one was used for scoring. The entire core(s) from each tumor was examined and at least 200 tumor cells had to be evaluable to be designated an intensity score. Positive staining was defined as complete and/or partial (>50% IR tumor cells) staining at any intensity that could be differentiated from truly negative staining, background and diffuse non-specific staining. Cytoplasmic staining in high-power fields (40X objective) was accepted as positive reaction.

Photographs were taken using a Zeiss Observer Z1 microscope and the Axiovision software (Carl Zeiss, Göttingen, Germany).

This material has been used previously and staining procedures and scoring of ER, PgR, Ki67, HER2 and Nottingham histological grade (NHG) of the material have been previously described^41^.

### Immunohistochemical controls

The specificity of the ghrelin and obestatin antibodies has been evaluated and presented previously^42^. Normal human gastric mucosa was used as positive control to assess the accurate immunoreactivity of the ghrelin and obestatin antibodies.

### Statistical analyses

The event was breast cancer-related death. Breast cancer-specific survival (BCSS) was defined as time from diagnosis until breast cancer-related death. Cox proportional regression was performed for the estimation of hazard ratios (HRs) and confidence intervals (CIs). The optimal cut-off values (IR (1 + 2 + 3) vs. non-IR (0) tumors) for ghrelin and obestatin in FBC have been determined previously^34^. However, the cut-offs may not be the same in MBC. In order to find the optimal cut-offs for ghrelin and obestatin, the HRs for breast cancer death for each cut-off was calculated, using cox proportional hazard model as previously described^34^. Briefly, the HR for different cut-offs were systematically analyzed. The optimal cut-off was defined as the value that best separated a poor prognostic group from a good prognostic group (i.e. the highest/lowest HR with a low p-value).

Established prognostic factors such as tumor size, age and histological grade, as well as ghrelin and obestatin were analyzed in univariate models. Spearman’s correlation test was used for correlations between variables. Kaplan-Meier plots were used for survival analysis, and the log-rank test was used for comparison.

A directed acyclic graph (DAG) was used to determine the appropriate factors to include in a multivariate model, indicating that none of the factors (ER, PgR, Ki67, age, node status, tumor grade, tumor size, HER2 and endocrine treatment) should be included in the model^43^. DAGs are frequently used to represent independence and relationships among random variables in a complex system^44,45^.

Comparison of the agreement of the two observers’ results was performed to evaluate the reproducibility of the scoring of the immunohistochemical results. The degree of concordance between the two investigators was quantified as kappa^46^. The commonly applied definition for the interpretation of different kappa values that was used here: 0.01–0.20 as none to slight; 0.21–0.40 fair; 0.41–0.60 moderate; 0.61–0.80 substantial; 0.81–1.00 as almost perfect agreement.

All statistical analyses were performed using IBM SPSS Statistics software (v25, USA).

## Results

### Immunoreactivity in breast tumor tissue samples

Results from routine stainings for hormone receptors and HER2 are described in the patients’ characteristics (Table 1). One hundred and eighty-nine and 186 cases with both tumor cores were available for immunohistochemical evaluation of ghrelin and obestatin, respectively. The remaining cases were either lost during technical preparation or did not contain a sufficient number of tumor cells (200 cells minimum). The array immunostained for ghrelin had 8 missing cases, and 7 cases with only one core, and the obestatin array had 11 detached cases and 7 cases with one core.

**Figure 1:**
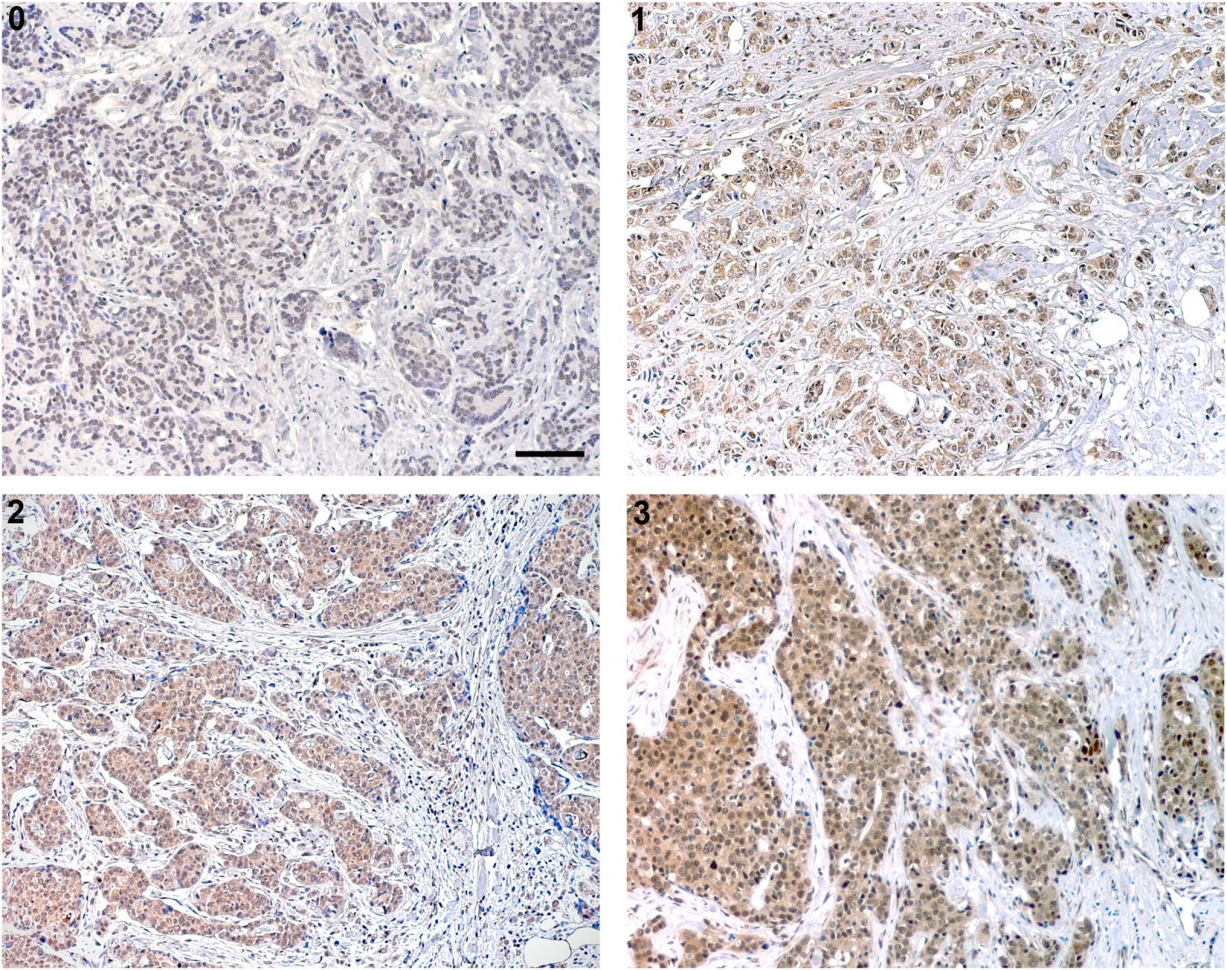
Ghrelin expression in male breast cancer tissue was analyzed by immunohistochemistry. Representative images of ghrelin with 0 (non-immunoreactive), 1 (weak), 2 (moderate) and 3 (strong) immunostaining. Scale bar = 100 μm.

Various patterns of immunostaining intensity were observed. Obestatin generally showed a stronger immunostaining pattern than ghrelin. In total, 179 of 189 (94.7%) evaluable cases immunostained for ghrelin showed the same degree of immunostaining intensity between the two core samples whereas this was true for 172 of 186 (92.5%) cases of the obestatin samples. Hence, the immunostaining score (from 0–3) was discordant between the two cores in 10 (5.3%) cases for the ghrelin immunostaining, whereas 14 (7.5%) of the obestatin cases were discordant. Of these, 6 (3.2%) ghrelin cases and 3 (1.6%) obestatin cases showed a major variance resulting in a difference between IR and non-IR within the same case. Most often the disconcordances within the same case were seen between score 1 and 2. Table 2 summarizes the results from the immunohistochemical scoring. Representative photos from the ghrelin immunostainings are shown in Fig. 1.

**Table 2:** Results from the immunohistochemical scorings.

	Ghrelin IR cases (%)	Obestatin IR cases (%)
Strong (3)	6 (3)	79 (40.1)
Moderate (2)	51 (25.9)	76 (38.6)
Weak (1)	103 (52.3)	17 (8.6)
Non-IR (0)	29 (14.7)	14 (7.1)
Missing	8 (4.1)	11 (5.6)
Total	197	197

IR, immunoreactive.

### Correlations to relevant clinicopathological parameters

Ghrelin and obestatin were positively correlated to each other (Spearman’s correlation). No strong correlations could be identified between ghrelin/obestatin and any of the investigated clinicopathological parameters (Table 3).

**Table 3:** Ghrelin and obestatin expression in male breast cancer in relation to clinicopathological variables.

Ghrelin	ρ	p-value	*n* (%)
Tumor size	0.04	0.58	175 (93)
Node status	0.04	0.66	158 (84)
Age	0.06	0.40	188 (99)
ER	0.20	0.007	186 (98)
PgR	0.15	0.05	186 (98)
HER2	−0.09	0.23	184 (97)
NHG	0.12	0.11	184 (97)
Ki67	−0.09	0.23	185 (98)
**Obestatin**			
Tumor size	0.04	0.62	172 (92)
Node status	0.03	0.74	155 (83)
Age	−0.05	0.54	185 (99)
ER	0.11	0.15	184 (99)
PgR	−0.25	0.001	184 (99)
HER2	0.06	0.38	182 (98)
NHG	0.05	0.55	181 (97)
Ki67	−0.12	0.1	183 (98)
Ghrelin	0.34	0.000	186 (100)

ER, estrogen receptor; HER2, human epidermal growth factor receptor 2; NHG, Nottingham histological grade; PgR, progesterone receptor; ρ, Spearman’s correlation test coefficient.

### The optimal cut-offs and association between the peptides and prognosis

The optimal cut-offs for ghrelin and obestatin corresponded to the previously defined optimal cut-offs in FBC (IR (1 + 2 + 3) vs. non-IR (0) tumors)^34^. Cut-offs corresponding to immunoreactivity scores of 0 + 1 vs. 2 + 3 and 0 + 1 + 2 vs. 3 were not statistically significantly associated with survival for neither ghrelin nor obestatin. Ghrelin had a HR of 0.68 (95% CI 0.34–1.37) and HR of 0.04 (95% CI 0–23.4), respectively. The same was true for obestatin with a HR of 0.57 (95% CI 0.24–1–38) for 0 + 1 vs. 2 + 3 and HR 1.20 (95% CI 0.64–2.23) for 0 + 1 + 2 vs. 3.

In univariate analyses, tumor size >20 mm, positive nodal status and expression of ghrelin were associated with breast cancer death. Any ghrelin expression was associated with prolonged BCSS with a HR of 0.39 (95% CI, 0.18–0.83) compared to lack of expression. Grade, Ki67, HER2 and age had HR:s over 1.0 but were not statistically significantly associated with survival, neither was obestatin, which was associated with HR below unity. The numerically highest HRs for breast cancer death were predicted by node status (HR ≥ 5). HR for breast cancer death using tumor size was numerically lower (HR < 3). Data is summarized in Table 4.

**Table 4:** Univariate and multivariate analysis of prognostic parameters of breast cancer death.

		HR (95% CI)	p-value
**Univariate analysis**			
Tumor size	>20 mm vs. ≤20 mm	2.83 (1.44–5.58)	<0.01
Node status	pN1-3 vs. pN0	5.54 (2.30–13.37)	<0.01
Ki67	≥14% vs. <14%	1.40 (0.71–2.60)	0.31
HER2	pos vs. neg	1.41 (0.55–3.65)	0.48
Tumor grade	III vs. I + II	1.49 (0.80–2.78)	0.21
Age	50–69 yrs. vs. <50 or ≥70 yrs.	1.07 (0.56–2.05)	0.84
Ghrelin intensity	0 vs. 1 + 2 + 3	0.39 (0.18–0.88)	0.01
Obestatin intensity	0 vs. 1 + 2 + 3	0.38 (0.11–1.24)	0.11
**Multivariate analysis**			
Tumor size	>20 mm vs. ≤20 mm	1.78 (0.85–3.72)	0.13
Node status	pN1-3 vs. pN0	4.52 (1.82–11.20)	<0.01
Ghrelin intensity	0 vs. 1 + 2 + 3	0.38 (0.17–0.88)	0.02

Hazard ratio (HR) and 95% confidence intervals (CI) obtained from Cox regression models. HER2, human epidermal growth factor receptor 2.

According to the DAG analysis, none of the factors influenced ghrelin in such a way that they should be included in a multivariate model. When adjusting for tumor size and node status, ghrelin was still a statistically significant prognostic factor with an HR of 0.38 (95% CI, 0.17–0.87).

**Figure 2:**
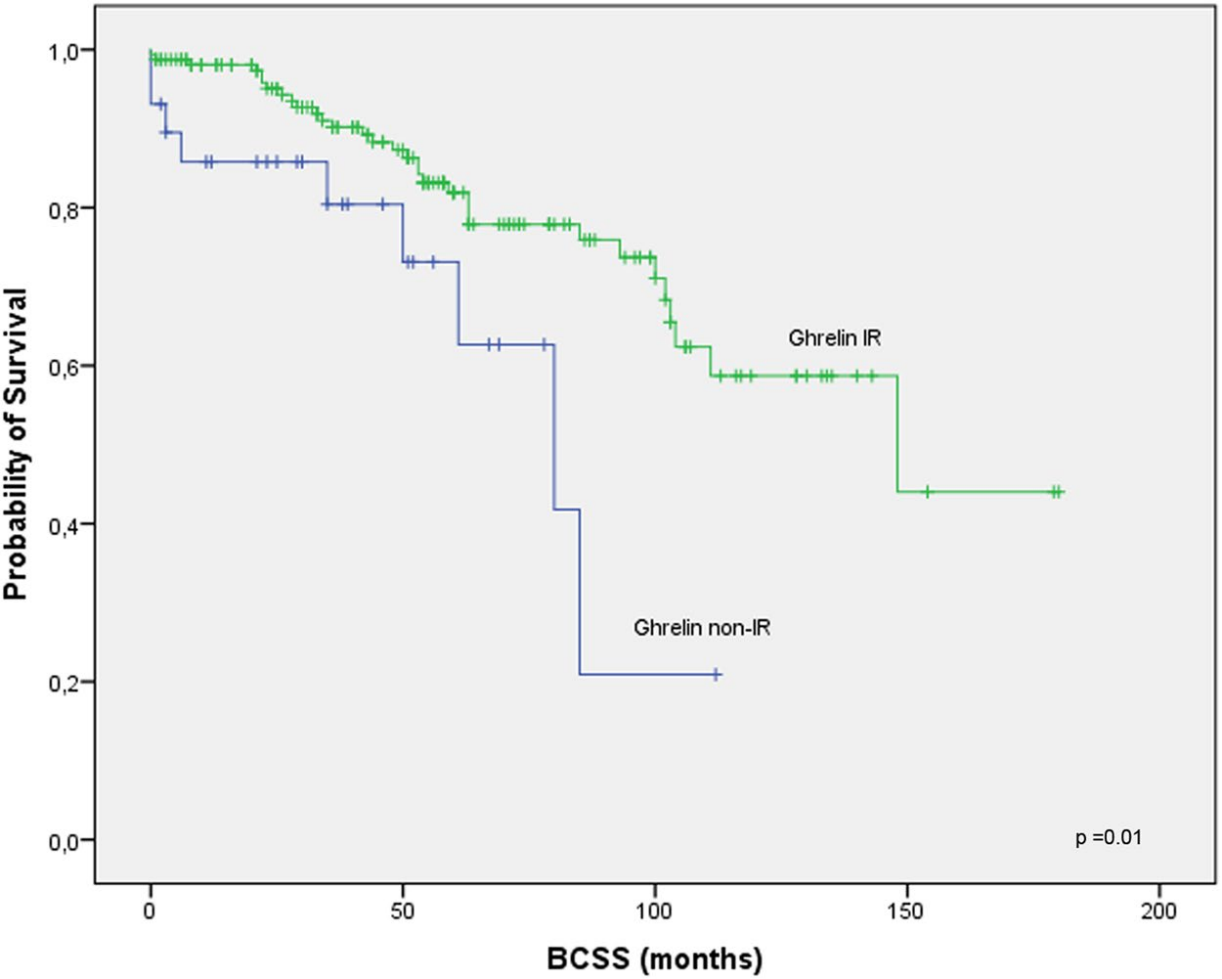
Survival among male breast cancer patients by ghrelin expression. Ghrelin immunoreactive, scores 1–3; ghrelin non-immunoreactive, score 0. BCSS according to ghrelin.

Kaplan-Meier analysis indicated that patients with tumors expressing ghrelin had a longer BCSS (Fig. 2, *p* = 0.01) compared to those with no ghrelin expression.

### Ghrelin and Endocrine Treatment

The predictive value of ghrelin in patients undergoing adjuvant endocrine treatment was analyzed. Sixty percent (*n* = 119) of patients underwent adjuvant endocrine treatment, and all but one (118 of 119) received tamoxifen. Endocrine therapy was administered more often in higher-stage disease: 79.5% in stage III, whereas only 46.9% in stage I. Patients with ER positive tumors undergoing endocrine treatment had a significantly poorer breast cancer survival compared with patients with ER positive tumors not receiving endocrine treatment. Ghrelin overexpression did not influence the response to endocrine treatment. An analysis of adjuvant endocrine treatment in patients with ER positive tumors adjusted for ghrelin did not result in any notable change of the HR for endocrine treatment; 2.34 (95% CI, 1.02–5.37) in the unadjusted vs. 2.24 (95% CI, 0.98–5.14) in the adjusted analyses.

### Reproducibility

The two investigators examined the ghrelin and obestatin immunostainings “blindly” and separately. The kappa value for ghrelin was 0.89 (almost perfect agreement) and for obestatin 0.65 (substantial), demonstrating very good reproducibility of the scorings.

### Control experiments

In all experiments the positive control for antibody accuracy showed ghrelin and obestatin IR cells in the deeper third part of the gastric mucosa as expected (data not shown).

## Discussion

In this study, the expression of ghrelin and obestatin was evaluated using immunohistochemistry in a well*-*characterized cohort of MBC cases, to assess their prognostic relevance. The results suggest that survival is better in MBC patients with tumors expressing ghrelin, than among patients whose tumors are non-IR. The levels of peptide expression were re-evaluated using different cut-offs but this did not demonstrate any prognostic value.

Although this is a large series of men with MBC with available tumor tissue and outcome data, the moderate statistical precision makes interpretation of statistically non-significant results difficult, and would therefore require validation in later studies. However, considering the rarity of this disease and also, compared with many other studies on MBC, this is a comprehensive study. In addition to reliable information on vital status and cause of death, access to clinicopathological and treatment data strengthen the study.

In order to assess if adjustment for a factor reduces or introduces bias and also to select any confounding factors where casual relations are described, DAG depicting causal relations was used. To the best of our knowledge, factors known to affect breast cancer survival such as ER, proliferation index and age, are not influenced by ghrelin in a way that it would affect breast cancer prognosis. Therefore, these factors should not be included in the statistical model. This is in agreement with the results that none of the factors included in the correlation analyses/DAG were strongly correlated to ghrelin.

The optimal cut-offs for ghrelin and obestatin may be different in MBC compared to FBC, and, in order to define the best cutoff, a previously described method was used^34^. The most optimal cut-off values for ghrelin and obestatin were in accordance with previous FBC material, indicating that there are no major differences in the prevalence of these marker expressions between male and FBC.

The cox regression analyses showed that ghrelin expression was associated with longer survival, where men whose tumors were IR for ghrelin had approximately 2.5 times lower risk for breast cancer-specific death. The analysis showed that apart from ghrelin, tumor size and nodal status were significant in terms of BCSS. Additional analyses to further exclude the risk of confounding showed that ghrelin was a prognostic factor independent from node status and tumor size. The results from the univariate analysis are in accordance with previously published results in MBC^39,47^. In earlier studies we have investigated ghrelin and obestatin expression in a large panel of invasive FBC^34^, as well as in lymph node negative breast cancer patients^35^ using the same technique and with similar results. In both studies, ghrelin expression was associated with a longer BCSS. Similar to this study, only ghrelin showed statistical significance in the regression analyses.

HR for obestatin was low, but with a wide CI. Due to the moderate number of events, the results of obestatin should be interpreted with caution, hence, a larger cohort may be needed to be able to establish its prognostic impact in MBC. The association between ghrelin and obestatin in this material was significant but weak (rho value 0.3) which could explain why the two proteins did not provide similar prognostic information. Even so, the result is in accordance with our previous studies^34,35^. The different peptide patterns within the same tumor could be due to alternative splicing of the ghrelin gene in different parts of the tumor. Furthermore, several possible post-translational cleavages sites may generate transcripts that encode new ghrelin gene-derived peptides that may act through different receptors and have novel biological functions^48,49^. Ghrelin gene-derived peptides may also be produced independently of preproghrelin^50^.

Several breast cancer studies have documented ghrelin and ghrelin gene-derived peptides^31,33–36,38^. However, comparable studies on ghrelin in breast cancer and especially MBC, and reports evaluating the prognostic role of ghrelin in breast cancer are still rare. Low levels of ghrelin has been demonstrated in normal breast tissue, with moderately higher levels of staining in breast cancer samples^33^. However, the potential prognostic role of ghrelin was not analyzed in that study. Another study, using qRT-PCR, reported that levels of ghrelin expression was equal between normal breast tissue and breast cancer. Interestingly, the ghrelin variant In1-ghrelin was overexpressed in breast cancer tissue, and in addition, it promoted basal proliferation in the breast cancer cell line MDA-MB-231^38^. This suggests that there is a different expression pattern of ghrelin and ghrelin-gene derived peptides and variants in normal versus breast cancer tissue, and that these variants may act differently on regulation and proliferation. It may therefore be of interest to evaluate other antibodies versus other transcripts in ghrelin system as well.

Using immunohistochemistry, we have demonstrated the presence of ghrelin and obestatin in normal female and male breast tissue, where the majority of samples expressed the peptides^32^. When comparing the immunohistochemical patterns in our previous studies on FBC^34,35^ with the present study on MBC, the expression of the peptides seem to be similar. The majority of the tumors express the peptides. A tendency towards a higher intensity in the obestatin stainings is seen in both MBC and FBC, whereas ghrelin is expressed more commonly in the low and moderate scoring scale.

*In vitro* studies have revealed that ghrelin both can inhibit and promote proliferation. In human breast carcinoma cell lines, ghrelin causes a decrease in proliferation and thus, the peptide may have a clinical application in breast cancer therapy^21–23,51^. In accordance with an inhibitory role of ghrelin in breast cancers, ghrelin expression correlates to low histologic grade, ER positivity, small tumor size, and low proliferation of human breast tumors^34^. However, also the opposite effect, where ghrelin promotes proliferation, has been observed^33^, hence, more *in vivo* studies are needed, since the use of cell lines and administrated doses of ghrelin may not represent physiological conditions.

In our study, patients receiving adjuvant endocrine treatment had a poorer breast cancer survival compared with cases without such treatment. The most plausible explanation for this finding is that treatment was given more often in higher-stage disease. However, the prognostic impact of adjuvant endocrine treatment did not change when adjusting for ghrelin, indicating that ghrelin expression does not influence the response to endocrine treatment in MBC.

The reproducibility of both peptide assessments in this study was good to very good with the kappa values 0.65 and 0.89 for obestatin and ghrelin, respectively. The stainings in the FBC^34^, node negative^35^ and MBC cohorts all had a good reproducibility and this strengthens the robustness of the evaluation. The evaluation separating only IR from non-IR tumors, was easily and quickly performed with a high reproducibility, an advantage for possible future use in the clinical setting.

There is an ongoing debate regarding the similarity between FBC and MBC, and if MBC may be a unique tumor type different from FBC^52–54^. Lobular carcinomas are not common in MBC (1%), but in FBC however, it is the second most common subtype (11.8%)^47,55^. Furthermore, MBC tumors are more often hormone receptor positive than FBC tumors, *HER2* appears to be less frequently over-expressed/amplified and differences in histologic grade have been reported^52,53,56,57^. Previous studies have demonstrated that different molecular profiles exist in MBC. MBC subgroups are different from FBC subgroups and MBC subtypes seem to differ in prognosis^12,41,58^. Immunohistochemical assessment to distinguish different subtypes using classifications validated in FBC may not be directly transferable to MBC. How to best define MBC subgroups by immunohistochemical markers, and if ghrelin/obestatin may be used to do this, remains to be elucidated. It may be of importance to analyze MBC as a separate disease and consequently, different markers may be of different importance in male and FBC. More studies will hopefully allow large-scale characterization and a better understanding of MBC biology. Men diagnosed with breast cancer may require other treatment strategies and management than FBC patients.

Interestingly, via the role of ghrelin, there is a novel link between obesity and breast cancer, since ghrelin inhibits aromatase expression in adipose stromal cells. Ghrelin is thereby able to regulate aromatase expression and activity^59,60^. Compared with FBC, MBC is almost always positive for ER^61^. Ghrelin analogues could therefore potentially be a therapeutic option of estrogen-dependent breast cancer.

In conclusion, the findings from this study suggest a potential role of ghrelin in breast cancer where ghrelin immunoreactivity corresponds to a better outcome. This finding, along with the easiness of ghrelin determination by immunohistochemistry, indicate that ghrelin may be used as a new marker for the prognostic assessment of MBC. Tumor size >20 mm and positive nodal status are also risk factors for breast cancer death in MBC. Obestatin, age, HER2, grade and Ki67 did not demonstrate any prognostic impact, but the moderate power of this study must be taken into account. Growing understanding of the functionality of ghrelin and the molecular pathways involved, also reveal a therapeutic potential for targeting ghrelin. Validation of the function of ghrelin in breast cancer in larger population studies is necessary, and should include functional and genetic studies.

## Data Availability

The datasets generated during and/or analyzed during the current study are available from the corresponding author on reasonable request.
